# Emerin oligomerisation properties, impact on lamin and actin recognition

**DOI:** 10.1186/1750-1172-10-S2-O17

**Published:** 2015-11-11

**Authors:** Isaline Herrada, Sophie Zinn-Justin

**Affiliations:** 1 Institut de Biologie Intégrative de la Cellule, CEA, CNRS, Université Paris Sud, Gif-sur-Yvette, France

## 

Since a few years, several studies have revealed the essential role played by the nuclear envelope in the cell response to mechanical demands of their immediate surroundings. A systematic scaling between the concentration of lamins within the nucleoskeleton and tissue elasticity was observed. This tuning in the nuclear lamina composition was associated to changes in nuclear mechanical properties. In addition to the lamin expression level, the phosphorylation states of lamins A and C and of their partner emerin might contribute to the transmission of a mechanical signal. In particular, in response to a force applied on nesprin-1 in isolated nuclei, emerin is phosphorylated on tyrosine residues Tyr74 and Tyr95, and these phosphorylation events are essential to trigger a nuclear mechanical response to tension. We have evaluated the capacity of the nucleoplasmic region of emerin to oligomerize, in the wild-type case as well as in emerin with mutations causing EDMD. We report large structural differences between wild-type emerin and several mutants. The impact of these structural differences on lamin and actin recognition is currently being studied. The role of emerin phosphorylation on emerin structure and binding properties is also being characterized, in order to reveal defects due to mutations causing EDMD.

